# Secure Computing for Fog-Enabled Industrial IoT

**DOI:** 10.3390/s24072098

**Published:** 2024-03-25

**Authors:** Ahmad Naseem Alvi, Bakhtiar Ali, Mohamed Saad Saleh, Mohammed Alkhathami, Deafallah Alsadie, Bushra Alghamdi

**Affiliations:** 1 Department of Electrical and Computer Engineering, COMSATS University Islamabad, Islamabad 45550, Pakistan; naseem_alvi@comsats.edu.pk (A.N.A.); bakhtiar_ali@comsats.edu.pk (B.A.); 2 Information Systems Department, College of Computer and Information Sciences, Imam Mohammad Ibn Saud Islamic University (IMSIU), Riyadh 11432, Saudi Arabia; maalkhathami@imamu.edu.sa (M.A.); 444008873@sm.imamu.edu.sa (B.A.); 3 Department of Computer Science and Artificial Intelligence, College of Computing, Umm Al-Qura University, Makkah 21961, Saudi Arabia; dbsadie@uqu.edu.sa

**Keywords:** secure computing, industrial IoT, trustable computing

## Abstract

Smart cities are powered by several new technologies to enhance connectivity between devices and develop a network of connected objects which can lead to many smart industrial applications. This network known as the Industrial Internet of Things (IIoT) consists of sensor nodes that have limited computing capacity and are sometimes not able to execute intricate industrial tasks within their stipulated time frame. For faster execution, these tasks are offloaded to nearby fog nodes. Internet access and the diverse nature of network types make IIoT nodes vulnerable and are under serious malicious attacks. Malicious attacks can cause anomalies in the IIoT network by overloading complex tasks, which can compromise the fog processing capabilities. This results in an increased delay of task computation for trustworthy nodes. To improve the task execution capability of the fog computing node, it is important to avoid complex offloaded tasks due to malicious attacks. However, even after avoiding the malicious tasks, if the offloaded tasks are too complex for the fog node to execute, then the fog nodes may struggle to process all legitimate tasks within their stipulated time frame. To address these challenges, the Trust-based Efficient Execution of Offloaded IIoT Trusted tasks (EEOIT) is proposed for fog nodes. EEOIT proposes a mechanism to detect malicious nodes as well as manage the allocation of computing resources so that IIoT tasks can be completed in the specified time frame. Simulation results demonstrate that EEOIT outperforms other techniques in the literature in an IIoT setting with different task densities. Another significant feature of the proposed EEOIT technique is that it enhances the computation of trustable tasks in the network. The results show that EEOIT entertains more legitimate nodes in executing their offloaded tasks with more executed data, with reduced time and with increased mean trust values as compared to other schemes.

## 1. Introduction

The emergence of smart cities represents a transformative shift in urban development, integrating advanced technologies to enhance the quality of life for residents and improve the efficiency of city operations. These cities leverage data and connectivity to optimize infrastructure, utilities, transportation, and public services. Smart cities deploy IoT devices, sensors, and data analytics to collect and analyze real-time information, enabling proactive decision-making and resource allocation. By fostering sustainable practices and citizen engagement, smart cities aim to address urban challenges such as traffic congestion, pollution, and energy consumption [1,2]. As these initiatives continue to evolve, smart cities are poised to drive innovation and economic growth and create more livable and resilient urban environments [3].

Smart cities encompass diverse applications empowered by a range of network providers. Thus, establishing trust in such a network becomes a fundamental challenge, necessitating the segmentation of networks across different infrastructures to accommodate diverse service providers and user types [4,5,6]. Additionally, the security of virtual machines is a concern, as they are prone to various types of attacks that can compromise network performance, seriously impacting the Quality of Service (QoS) [7,8]. There is also a risk that third-party-deployed virtual networks may become infected during operation, leading to communication disruptions [9,10].

The Internet of Things (IoT) plays a pivotal role in different smart city applications. IoT-based Industrial (IIoT) optimizes operations by collecting real-time data with the help of IoT sensors. IIoT enables predictive maintenance [11], asset tracking [12], and process automation [3]. IoT devices with the assistance of data monitoring tools can provide smart management of logistics [13]. Furthermore, data from IoT-based sensors help in the smooth operation of equipment by predicting necessary maintenance to minimize downtime by avoiding equipment failure. IIoT allows remote monitoring and control of industrial processes, equipment, and systems, enabling operators to manage operations from anywhere [14].

**Figure 1:**
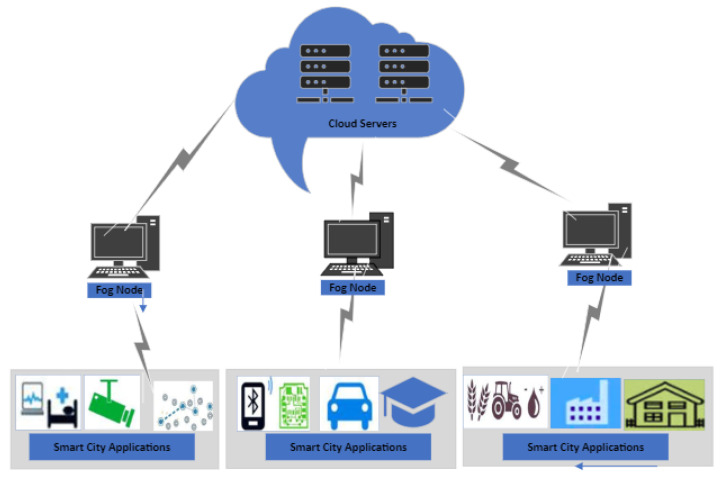
Architecture of fog-enabled Smart City infrastructure.

IoT nodes comprise sensor nodes that have limited processing and storing capacity [15,16]. In IIoT, sensor nodes are required to perform different types of tasks and sometimes have to run complex algorithms. Due to limited processing capacity, the sensor nodes may take longer time to execute these tasks which might not be possible for some time-constrained tasks. To execute these tasks within a specified time frame, these tasks are required to be offloaded for faster execution. Offloading tasks to more powerful machines helps in optimizing the use of resources. Furthermore, offloading latency-sensitive tasks in IIoT applications can achieve faster response times, which is crucial for real-time applications. Fog computing nodes provide data storage and computing services to the Industrial IoT nodes [17,18]. They are CPU servers that have better processing capacities than the IoT nodes. Nodes that facilitate fog computing are preferably placed in different areas at the edge of the different IoT networks in smart cities for increased storing and processing capacity [19,20,21] as shown in Figure 1. These fog nodes can be used for efficient task execution of these IIoT nodes. There are many industrial fog node products that are used in real IoT environments [22].

Advancement of diverse communication technologies, such as Sixth Generation (6G) cellular communications and Low Power Wide Area Network (LoRaWAN), enables IoT nodes to adapt to these technologies for efficient working of smart city applications. In IIoT, nodes are wirelessly connected and chances of vulnerability increase due to diverse heterogeneous wireless networks and chances of malicious attacks increase. Malicious attacks may create anomalies in the communication process in different prospects and result in a compromised QoS of the network. Malicious node attacks may also cause delays in executing the offloaded tasks by fog nodes by offloading the time-consuming complex tasks to fog nodes. These malicious node tasks utilize the computing resources of fog nodes, causing delays in executing the tasks offloaded by the legitimate nodes.

To handle the above challenges for improved performance of the fog computing nodes, the offloaded tasks anomaly is required to be resolved by differentiating the malicious and legal nodes’ tasks. Tasks offloaded by malicious nodes affect the fog node’s performance in executing legitimate tasks due to the limited processing and storing capacity of the fog node and sometimes can not execute all the offloaded tasks within their stipulated time duration. In this paper, an Efficient Execution of Offloaded IIoT Trusted Tasks (EEOIT) mechanism for secure and reliable computing is proposed. EEOIT assists fog nodes in executing most of the tasks transmitted by valid and trustable nodes in its processing cycle by applying the Technique for Order Preference by Similarity to Ideal Solution (TOPSIS) [23].

The salient features of the proposed EEOIT include:

1. A novel trust management scheme to prevent anomalies caused by security attacks and differentiate the trustable tasks from the malicious tasks.
2. An efficient TOPSIS-based offloading task priority mechanism for all offloaded trusted tasks within the task deadline.

The organization of the paper is structured as follows:

Section 2 discusses prior research endeavors, specifically exploring trust management from various perspectives. An overview of the system model and details of our proposed schemes are mentioned in Section 3 and Section 4, respectively. Section 5 conducts a comparative analysis through extensive simulations and presents the results. Finally, Section 6 serves as the conclusion, summarizing key findings and concluding our manuscript.

## 2. Related Work

There have been several techniques that have worked on securing communications and computing in smart city-enabled IoT. Numerous studies have explored trustful communication in various communication domains.

The work in Ref. [24] proposed the MATS framework by using a game theoretical approach to solve the issue of trustful communications. Moreover, the framework also considers different situations and possible malicious issues for each scenario. The proposed technique presents a dynamic trust solution that works for multiple scenarios. Experimental results validate the framework’s performance in their work.

In Ref. [25], authors considered an Unmanned Aerial Vehicle (UAV) scenario that can assist IoT-based Intelligent Transportation Systems (ITS). The proposal provides a trustable data collection scheme that also considers data deadlines. Furthermore, the trajectory of UAVs is also optimized so that trustable communications can be enhanced. Results provide reduced delays and costs by the developed system.

Authors in Ref. [26] highlighted the scenario of online social networks where trust is a major challenge and proposed a trust aware framework for online networks. The proposed technique provides a mechanism to handle malicious nodes in the network. The trust model is developed to assign a trust value to each user. Moreover, the work also provided a data balancing technique. The simulation results highlight the improvement achievement in terms of trust and data precision.

In Ref. [27], authors proposed a MapReduce-based Framework for the management of big data along with handling trust. MapReduce-based framework focused on big data problems for data processing. A trust framework is developed to handle the scheduling of MapReduce. Results show the significance of the proposed technique for managing trustable big data.

Authors in Ref. [28] proposed a trust management for online systems that focused on online web-based trust challenges and considered a virtual network scenario. Particularly, the focus of the work is on handling the issues of virtual networks during their running time as well as when the system reboots. Furthermore, the work also developed trust management procedures. The work also implemented a prototype using an open-source MANO platform and evaluated network performance in a dynamic environment.

In Ref. [29], an IoT-based healthcare system with a decentralized trust management system for secure and distributed healthcare is explored. To implement trust-based communication in the network, an evidence–theory-reliant solution is proposed. A reward and punishment system is established to manage trust-based data communications. The performance analysis of the proposed technique shows robustness and efficiency with security against various types of attacks.

In Ref. [30], authors focused on how technicians can easily and confidently intervene on industrial equipment with the joint adoption of new technologies. In this work, the authors describe the design of a software architecture aimed at simplifying the management, configuration, and assessment of IIoT systems. Furthermore, they discuss their experiences with the proposed architecture in a railways use case.

Authors in Ref. [31] addressed the management difficulty faced by such data owners’ authority that depends on a Trusted Third Party (TTP) by applying key aggregate searchable encryption (KASE). The authors proposed a secure data-sharing system based on KASE in a fog-enabled IoT environment using blockchain and applied Burrows–Abadi–Needham (BAN) logic. The authors claimed that their proposed scheme guaranteed secure mutual authentication.

The authors of [32] have identified the conventional real-time security concerns that end-users face in an IoT network. They have proposed a layered architecture within the fog computing paradigm to address these issues. Furthermore, they have explored a range of existing solutions that have been proposed to overcome these real-time security challenges.

A summary of all the references discussed in this section are represented in Table 1.

## 3. System Model

**Figure 2:**
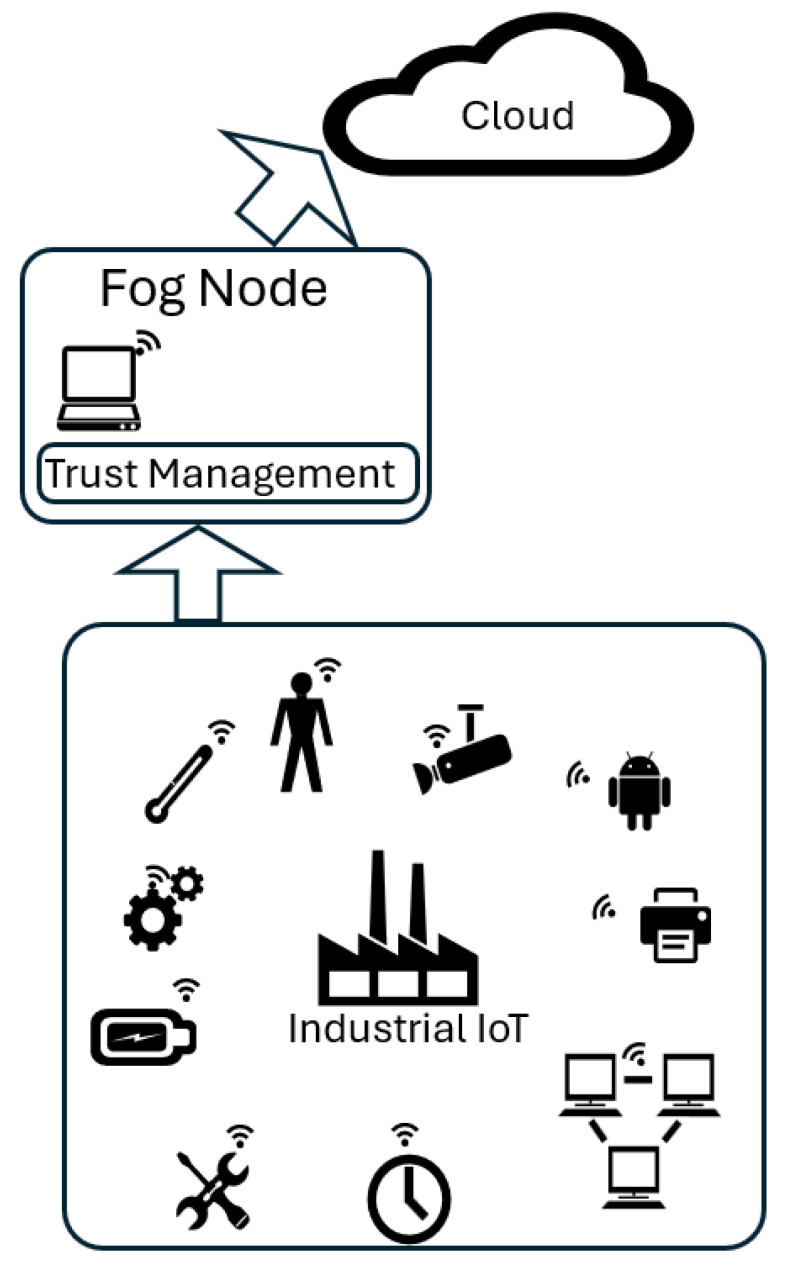
System model.

The IoT-based industrial applications demand varying computing and processing tasks. As the computing capability of these nodes is not high, these tasks can not be processed in the required time frame and must utilize nearby fog servers. These tasks to be offloaded are different in size and associated computing requirements. The fog node receives a different number of offloaded tasks in different time intervals. There are chances of malicious attacks in the network that may create anomalies in the network by offloading tasks that may require a higher processing time; while the fog server has higher capacity than the IIoT node, it still has a limited computing space, and if the offloaded tasks are more than its processing capacity, these will be forwarded to the cloud servers for processing. The considered system model is shown in Figure 2.

IIoT nodes are directly connected with fog nodes. The fog node after regular time intervals monitors the tasks at their input. The monitoring time is calculated as the maximum time ($t_{max}$) required by a node in transferring its task to the fog node. In this IIoT network, there are *N* nodes attached to the fog node that is a combination of *L* number of trustable nodes and *M* number of malicious nodes. A fog node receives the trust values from all the attached IIoT nodes within its $t_{max}$. The fog node after each $t_{max}$ time interval re-assesses the values of trust for all attached nodes.

Suppose the number of tasks sent for computation by user *A* is $T_{i}$, and *K* out of the available *N* number of nodes offloaded one or more tasks to the fog server. The sum of all offloaded tasks $\sigma_{Tot}$ at the fog node after *M* number of processing intervals is calculated as

(1) $$\sigma_{Tot}=\sum_{i=1}^{K}\sum_{j=1}^{M}T_{ij}$$

If the data size of task $T_{i}$ is $D_{i}$ and the computation capacity for processing tasks of the fog node is $P_{B}$, then the total amount of data executed ($\theta_{Tot}$) by the fog node along with the processing time ($\zeta_{Tot}$) required by the fog node to execute $\sigma_{Tot}$ tasks is calculated as mentioned in Equations (2) and (3), respectively.

(2) $$\theta_{Tot}=\sum_{i=1}^{K}\sum_{j=1}^{M}D_{ij}$$

(3) $$\zeta_{Tot}=\sum_{i=1}^{K}\sum_{j=1}^{M}\frac{D_{ij}}{P_{B}}$$

Suppose, out of the *N* number of nodes, there are NT non-trusted nodes in the network, and out of these NT nodes, *H* number of nodes offloaded their tasks with data $D_{H}$, then the total legal offloaded data $\theta_{NT}$ computed by fog node in *M* number of sessions is calculated as

(4) $$\theta_{leg}=\sum_{i=1}^{K}\sum_{j=1}^{M}D_{ij}-D_{H}$$

The task computation time for all trustable nodes $\zeta_{leg}$ is given by

(5) $$\zeta_{leg}=\sum_{i=1}^{K}\sum_{j=1}^{M}\frac{D_{ij}-D_{H}}{P_{B}}$$

## 4. Proposed EEOIT Scheme

This research introduces a trust management framework designed for fog computing nodes to effectively handle the execution of offloaded tasks from various smart city applications. The scheme addresses the anomaly posed by malicious nodes, which attempt to jeopardize the performance of the fog node by submitting tasks that utilize its resources for execution, ultimately compromising the system’s integrity. In this work, an Efficient Execution of Offloaded IIoT Trusted Tasks Mechanism (EEOIT) for fog nodes is proposed. EEOIT provides a trust management system to avoid anomaly attacks by distinguishing between malicious and legitimate nodes. Furthermore, the proposed scheme offers an efficient algorithm for executing tasks on fog computing nodes. The main features of the proposed schemes are

- A combination of both direct and indirect trust is used for trust evaluation for all directly attached IIoT nodes.
- Task computation at the fog nodes is managed by using the TOPSIS value of input tasks based on different parameters.

### 4.1. Trust Management

Malicious attacks disturb the QoS of the network. In IIoT networks, nodes offload their tasks to nearby fog nodes. Malicious nodes compromise the processing capacity of the fog node by uploading such offloaded tasks to fog nodes that require more execution time. The problem can be resolved by identifying the legitimacy of the tasks. In this section, the trust value of the offloaded tasks is calculated, which helps a fog node in differentiating the trust value of the offloaded task nodes.

#### 4.1.1. Trust Calculation of Directly Connected Nodes

The trust value of each node present in the IIoT network is calculated with the help of their neighboring nodes. Each node evaluates the trustfulness of other nodes in the network by exchanging its information with other nodes. Suppose node *A* receives *P* packets from its neighboring node *B* during the last periodic cycle. Out of these *P* packets, $P_{c}$ packets are error-free and correctly received by node *A*. To calculate the trustworthiness of node *B* ($T_{B}^{A}$), node *A* considers the previously calculated trust value for the last *N* packets and applies the following formula:

(6) $$T_{B}^{A}=\frac{\left(T_{B}^{A}\times P\right)+\left(P_{c}\times N\times SNR_{B}^{A}\right)}{2P\times SNR_{max}}$$

Here, $SNR_{B}^{A}$ represents the signal-to-noise ratio between node A and node B which is given as $\mathrm{SNR}=\frac{\mathrm{Signal}\;\mathrm{Power}}{\mathrm{Noise}\;\mathrm{Power}}$. The SNR gives us a measure of link quality among the nodes. All the trust values calculated are in the range of 0 to 1.

#### 4.1.2. Trust Calculation by Most Trusted Nodes

A node calculates the trust value of such a node that is not in its direct access through an indirect method by obtaining the information forwarded by the most trusted intermediate nodes. Suppose a node *A* wants to know the trust value of node *F* that is at the second hop distance from it. The trust value of node *A* for trust finding node *F* through most trusted nodes ($T_{MTN}^{F}$) with the help of the intermediate node *B* is calculated as

(7) $$T_{MTN}^{F}=\frac{T_{B}^{A}+T_{F}^{B}}{2}$$

**Figure 3:**
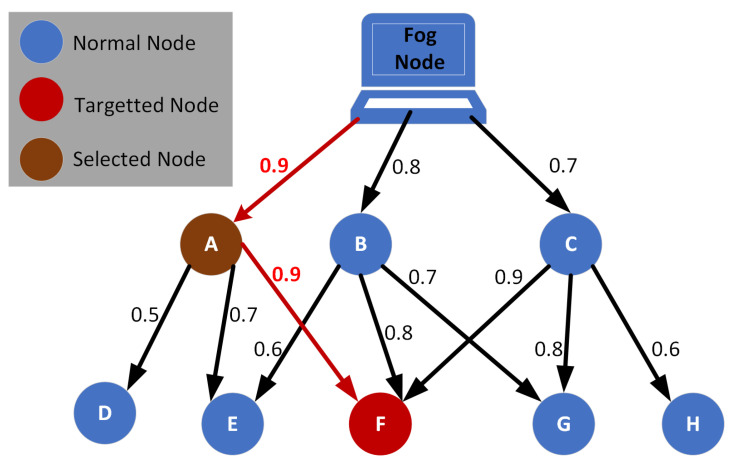
Nodes path selection through most trusted nodes.

When multiple trustworthy nodes are connected directly to the source node that is linked with the trust-finding node, the directly connected nodes with the highest trust value will be given preference. Suppose there is a fog node that needs to determine the trust value of a node *F*. Node *F* is directly connected to nodes *A*, *B*, and *C*, which have trust values of 0.9, 0.8, and 0.9, respectively. The fog node has calculated the trust values of nodes *A*, *B*, and *C* to be 0.9, 0.8, and 0.7, respectively, as shown in Figure 3. In this scenario, the trust value of node *F* will be computed through node *A* because it has the highest trust value among all three nodes. If there are multiple directly connected nodes with the same trust value, the node with the highest trust value towards the target node will be selected. This increases the chances of finding the legitimate end node, as trustworthy nodes will pass on all relevant information.

#### 4.1.3. Indirect Trust Calculation

The trust value of a trust-finding node calculated by all its neighboring nodes is also taken into consideration and is calculated by using an indirect trust calculation method. Indirect trust is calculated as the average sum of trust values collected in favor of a trust-finding node by all its neighboring nodes in the network.

Suppose there are *X* number of neighboring nodes of a trust finding node *F*, then the indirect trust calculation by all its neighboring nodes ($T_{ind}^{F}$) is calculated as

(8) $$T_{ind}^{F}=\frac{\sum_{i=1}^{X}T_{i}^{F}}{X}$$

#### 4.1.4. Trust Calculation

The updated trust value of each node for its associated node is forwarded to the fog node after regular time intervals. The calculated trust value in the proposed scheme is determined by the following methods:

1. The trust value calculated by the fog node about its directly connected nodes through their self-calculated trust value as mentioned in Equation (6).
2. The trust value calculated by a node about another node through such directly connected nodes which have highest trust values as mentioned in Equation (7).
3. Information about trust obtained from other nodes in the network for a node that is neither directly connected nor through the best-trusted nodes.

The trust value of any node in the network is calculated by assigning different weights to the trust values collected in the above-mentioned three methods such as

- The highest weights are assigned to the trust value collected by the fog node directly.
- Medium weights are assigned to the trust value collected by the nodes that arrive through the most trusted intermediate nodes as mentioned in Section 4.1.2.
- The least weights are assigned to the trust value determined through the indirect methods as mentioned in Section 4.1.3.

The trust probability value of a trust finding nodes TF in a network is calculated with the help of a sigmoid (η) function as mentioned in Equation (9).

(9) $$\sigma TF=\frac{1}{1+e^{-[H\left(T_{B}^{TF}\right)+M\left(T_{MTN}^{TF}\right)+L\left(T_{ind}^{tf}\right)]}}$$

**Figure 4:**
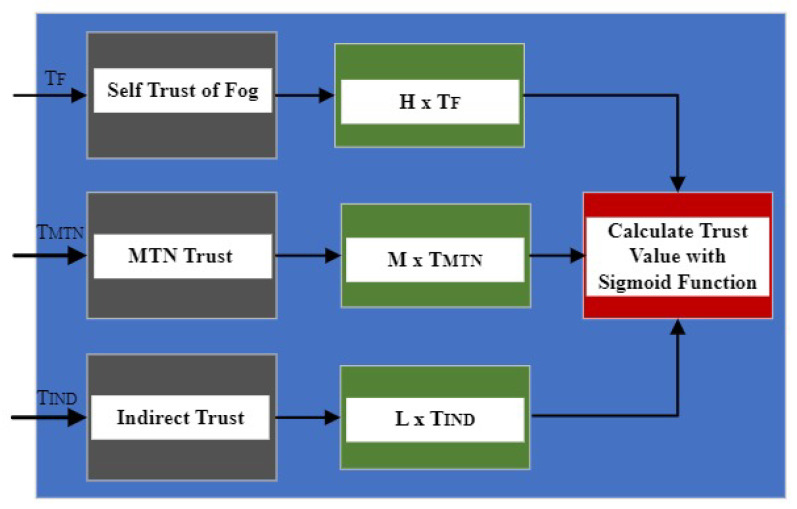
Trust calculation procedure for legitimate nodes.

Here, H, M, and L are the weights added to the different trust values calculated for the trust finding node TF with *H* representing the highest weight, *M* representing the medium weight, and *L* representing the least weight. The trust evaluation mechanism is depicted in Figure 4.

Fog servers after computing the trust value of each node present in the network perform a comparison with a threshold. If the trust value calculated is higher than the threshold value then it is considered as a legitimate node. However, if the trust value is less than the threshold value, then it is considered a malicious node. A complete procedure in differentiating the legitimate and malicious nodes is represented in the Algorithm 1.
**Algorithm 1:** Algorithm for Legitimate Nodes Detection
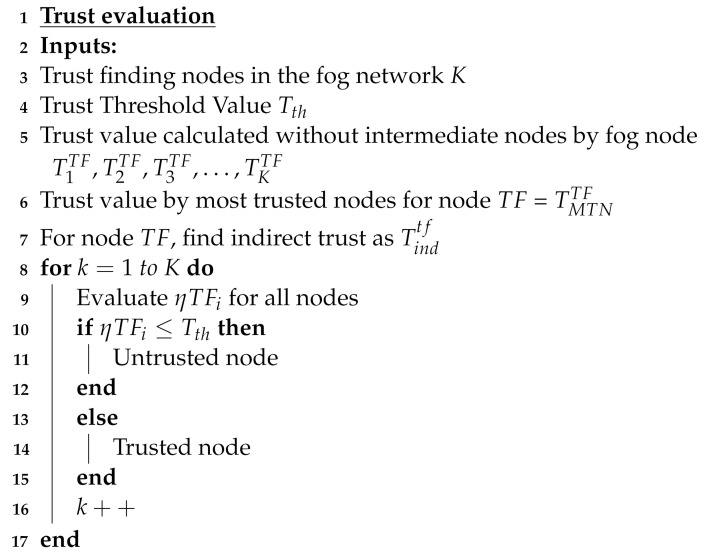



### 4.2. Proposed Task Execution Mechanism

The fog node, after receiving all the tasks offloaded by the IIoT nodes, calculates their capacity to execute them. After this, the fog node scrutinizes the legitimate tasks after discarding the tasks offloaded by malicious nodes using the proposed mechanism. If the offloaded legitimate tasks received by the fog nodes are within the processing capacity of the fog node, then all the offloaded tasks will be executed. However, if the offloaded tasks are more than the processing limit of the fog node, then it has to scrutinize the offloaded tasks before execution by applying a Technique for Order Preference by Similarity to the Ideal Solution (TOPSIS).

TOPSIS is a multi-criteria technique that provides the solution based on the TOPSIS score. The TOPSIS score is calculated to find the preference list based on the preference list provided to it. In this work, the TOPSIS is used to scrutinize the offloaded tasks that are required to be executed by the fog node. The tasks with the highest TOPSIS value are selected within the processing capacity of the fog node. The TOPSIS value in scrutinizing the offloaded tasks is based on the following parameters.

1. Trust Value of Tasks The trust is an important parameter in scrutinizing the tasks to be executed. The higher the trust value of the task, the higher the preference for the task.
2. Task Size The task size is also considered in such a way that the higher the size of the tasks, the more will be their preferences.
3. Task Elapsed Duration The task deadline is another parameter that is taken into consideration in computing the TOPSIS value of the task. The shorter the deadline of the task, the higher its preference will be.

Considering the above-mentioned parameters, the TOPSIS value is calculated against all the received tasks offloaded by legitimate nodes. After applying the TOPSIS algorithm, a TOPSIS value for each legitimate task is calculated. The tasks with the highest TOPSIS values are selected for the execution process. If there is still room for the tasks to be placed in the queue, then the next highest TOPSIS value tasks are scrutinized. The process continues until the processing capacity of the fog computing node is reached. A complete task scrutiny mechanism for execution of offloaded tasks is shown in Algorithm 2.
**Algorithm 2:** Scrutiny of Offloaded Task Algorithm using TOPSIS1**Inputs:**2Parameters used for task scrutiny (Trust probability $\sigma_{TF}$, task size $t_{s}$, time elapsed *t*)3**Steps of Algorithm:**41: Normalization of individual vectors per task offloading feature ($\sigma_{TF}$, $t_{s}$, *t*). The Objective of this algorithm is to maximize all the attribute values for the offloaded tasks.5    $\mathrm{Normalized}\;\mathrm{Value}=\frac{\mathrm{Actual}\;\mathrm{Value}}{\sqrt{\sum \mathrm{Actual}\;{\mathrm{Value}}^{2}}}$62: Calculation of the weighted normalized values for each offloaded task7    WeightedValue = $\left(\sigma_{TF}\times W_{\sigma_{TF}}\right)+\left(t_{s}\times W_{t_{s}}\right)+\left(t\times W_{t}\right)$8    where $W_{\sigma_{TF}}$, $W_{t_{s}}$, and $W_{t}$ are the weights.93: Calculation of the negative-ideal and ideal solutions for each feature.10     For the Ideal solution, use the Maximum normalized value for all the parameters.11     For the Negative-ideal solution, use minimum normalized value for all the parameters.124: Calculation of the nearness of every task to the negative-ideal and ideal solutions using a Euclidean distance measure.13     $D_{i}^{+}=\sqrt{\sum_{j=1}^{m}{\left({\mathrm{NormalizedValue}}_{ij}-{\mathrm{IdealSolution}}_{j}\right)}^{2}}$14     $D_{i}^{-}=\sqrt{\sum_{j=1}^{m}{\left({\mathrm{NormalizedValue}}_{ij}-{\mathrm{NegativeIdealSolution}}_{j}\right)}^{2}}$15**Step 5: Computation** of the TOPSIS measure for every task:16     $TOPSIS_{i}=\frac{D_{i}^{-}}{D_{i}^{+}+D_{i}^{-}}$17**Step 6: Ranking** of the tasks based on their TOPSIS scores.18     Tasks with a greater value of TOPSIS will be the most favorable for offloading as they match the criteria of higher value for all the parameters.19**Output:**20The prioritized offloaded tasks for offloading based on TOPSIS scores.21Task offloading starting from highly ranked tasks based on TOPSIS.

## 5. Comparative Results and Discussion

In this section, the EEOIT is compared and analyzed by creating a simulation environment in MATLAB R2023a. In this simulation environment, IIoT nodes are deployed over a region of 10×15 m with diverse sizes of executable tasks having different deadlines. Each node will have a particular trust value which will represent if the node lies in the legitimate or a malicious category. Depending on the trust value, the nodes mainly comprise legitimate nodes and some malicious nodes and their tasks are offloaded to the fog node. The offloaded tasks are in the random range of 10 to 30 with a diverse data range of 50 kB to 100 kB. The task computing capacity of the fog node ranges from 10 to 30 cycles and each of the executed tasks is calculated in terms of processing cycles. The main parameter values that are used in this simulation environment are mentioned in Table 2. We carried out Monte-Carlo simulations of over a 1000 iterations to calculate the average value of all the parameters.

EEOIT is analyzed and compared with three well-known standards Random [33], Shortest Job First (SJF) [21], and Longest Job First (LJF) [34] algorithms. The results regarding the number of offloaded tasks executed, total executed data, and mean trust value of legal tasks are obtained. These results are analyzed for varying numbers of nodes and varying computational capacities of fog servers with trust thresholds of 0.4 and 0.5.

### 5.1. Execution of Legitimate Tasks

The offloaded tasks are supposed to be executed successfully if it is executed within their defined deadline. The execution of offloaded tasks depends upon the offloaded tasks received along with their size and computational capability of the fog node in executing the tasks in a processing cycle for threshold values of 0.3 and 0.5.

**Figure 5:**
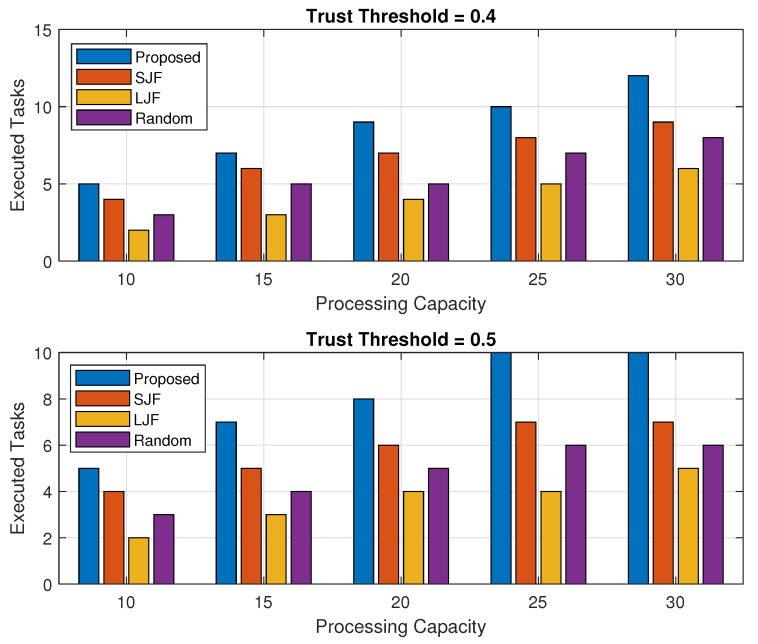
Executed legal tasks against fog processing capacity.

**Figure 6:**
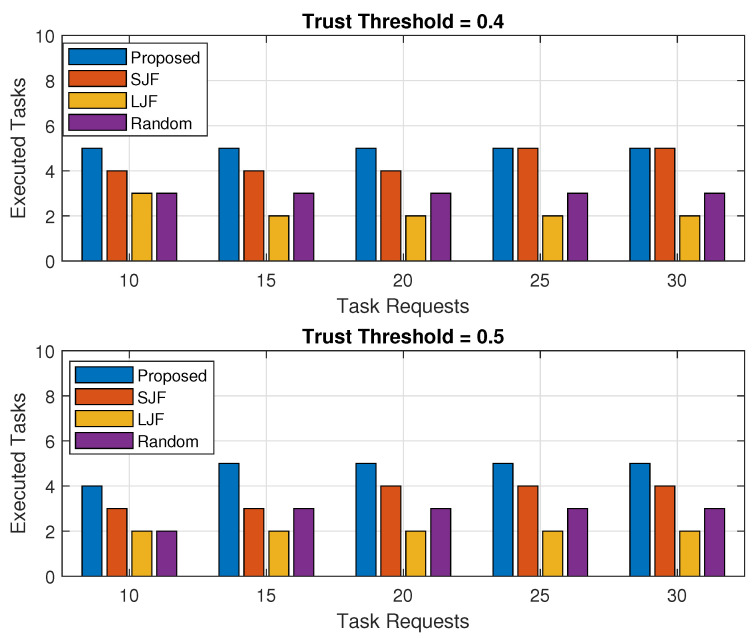
Executed legal tasks against varying number of task requests.

**Figure 7:**
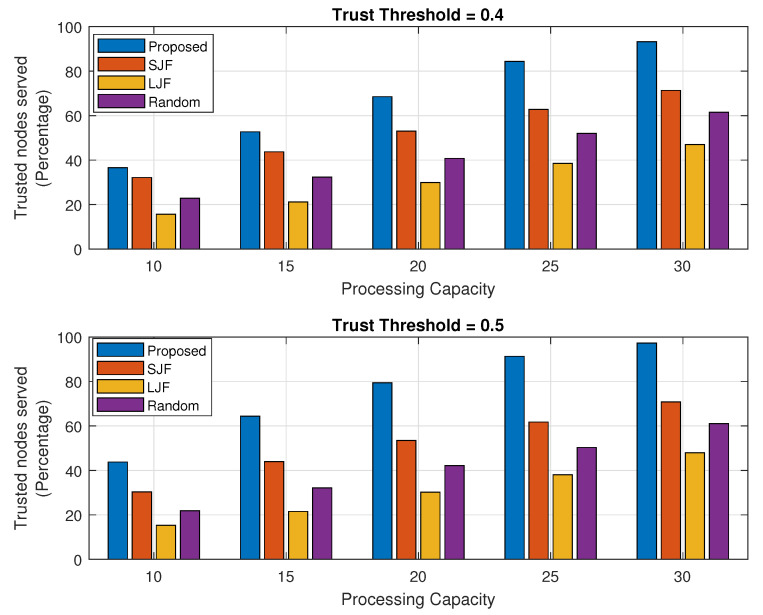
Percentage of executed legal tasks against fog processing capacity.

**Figure 8:**
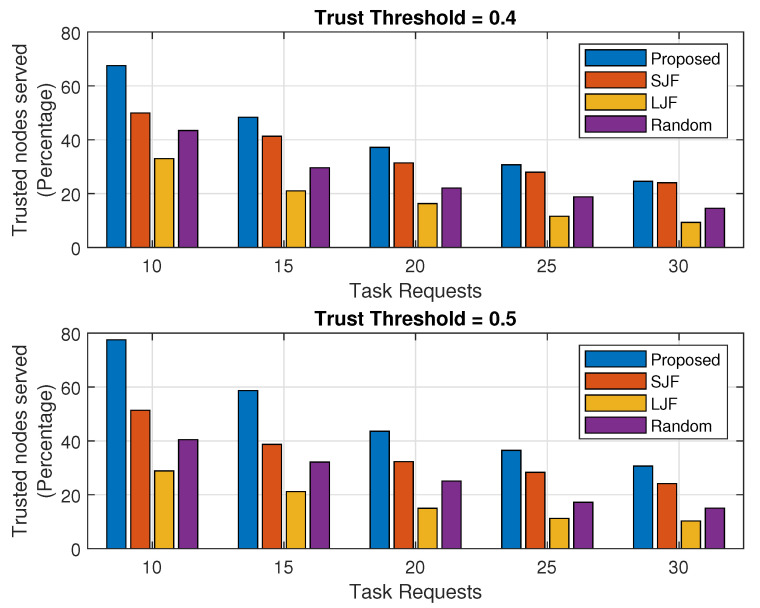
Percentage of executed legal tasks against varying number of task requests.

In this section, the successful execution of legitimate tasks for the fog node’s varying computing capability and an increasing trend of task requests received by the fog node with fixed processing capacity are discussed as mentioned in Figure 5 and Figure 6, respectively. To obtain a better picture of these results, they are also represented in terms of percentage as mentioned in Figure 7 and Figure 8.

Results in Figure 5 are a combination of two sub-figures that are obtained for trust threshold values of 0.3 and 0.5. The results show that for both of the trust threshold values, the fog node executes more offloaded tasks in the proposed scheme in comparison to all compared schemes against all varying computing capabilities of fog computing machines because EEOIT scrutinizes the legitimate nodes’ tasks first. In addition, the TOPSIS algorithm helps the fog node in executing more tasks within the same processing cycles. It has been observed from the results that the number of tasks executed by the fog node increases with its increased processing capability. For a threshold level value of 0.4, more offloaded tasks are considered legal nodes as compared to the threshold level of 0.5, and consequently, EEOIT executes more offloaded tasks for a threshold value of 0.4 as compared to 0.5 threshold values. It has also been observed from the results that the number of executed task requests by SJF is more than Random and LJF in both sub-figures as SJF allows the fog node to execute those tasks that are shorter in size, resulting in more tasks to execute within the specified processing cycle.

The same trend follows for a varying number of offloaded tasks when the number of offloaded tasks is increased by fixing the fog node’s processing capacity as shown in both sub-plots of Figure 6. It is evident for both threshold values of 0.4 and 0.5 that the number of offloaded tasks executed by the fog node in EEOIT is higher than for all the compared schemes. It has been observed from the results that for a threshold value of 0.4, more offloaded tasks are considered as legal as compared to a threshold value of 0.5. The higher the trusted tasks, the more the tasks will be executed by SJF, LJF, and Random. However, the number of executed tasks in EEOIT is the same when task requests increase because the fog node has already executed the maximum number of tasks. The results further show that the task requests entertained in LJF are the minimum among all, because, with the increased number of tasks, the number of larger tasks increases, and a smaller number of larger tasks will be executed in a specified time.

The percentage of the executed tasks is observed for an increasing trend of computing capability of the fog machine as well as for an increasing number of received tasks as shown in Figure 7 and Figure 8, respectively. Each of the results is a combination of two sub-plots with trust threshold values of 0.4 and 0.5. The percentage is determined by calculating the total number of legitimate tasks executed by the fog node against the legitimate task requests of IIoT nodes. It is evident from the results in both sub-figures that the percentage calculated for the executed tasks in the proposed EEOIT algorithm is more than the other three schemes. Results in Figure 7 represent that the increased task computing capability of the fog machine improves the execution percentage of task requests for both trust threshold values of 0.4 and 0.5. The fog node executes more tasks against the fixed number of 10 offloaded tasks in all processing cycles, and the percentage of executed tasks increases from 37% to 90% when the trust threshold is 0.4 and from 42% to 96% for the trust threshold value of 0.5 for the same amount of tasks requests. The same trend follows in all the compared algorithms as the executed tasks percentage improves with the rise in fog computing capability. However, the maximum percentage of the executed tasks in SJF, LJF, and Random are 72%, 48%, and 62%, respectively, for both the threshold values of 0.4 and 0.5.

For varying numbers of received task requests, the executed tasks percentage in EEOIT is more than the other three schemes for both trust threshold values as shown in both sub-plots of Figure 8. It is emphasized from the results that an increased number of task requests reduces the executed tasks percentage as the fog machine holds the same computing capability and can execute only a limited number of offloaded tasks, and consequently, the percentage of the executed tasks reduces. The results show that when the threshold value is 0.4, the executed tasks percentage reduces from 67% to 23% as compared to 52% to 23% in SJF, 35% to 8% in LJF, and 42% to 16% in Random. The results further show that for a threshold value of 0.4, there will be more offloaded trusted nodes in different sizes. For a threshold value of 0.5, the number of legitimate nodes in the offloaded tasks is less as compared to the threshold value of 0.4. For a threshold value of 0.5, the executed tasks by the proposed scheme are more than the other three schemes for fixed processing capacity and consequently an increase in the percentage of the executed tasks. The results show that the task execution percentage in the proposed scheme decreased from 78% to 32% as compared to 50% to 24% in SJF, 30% to 8% in LJF, and 40% to 15% in Random.

### 5.2. Executed Data

**Figure 9:**
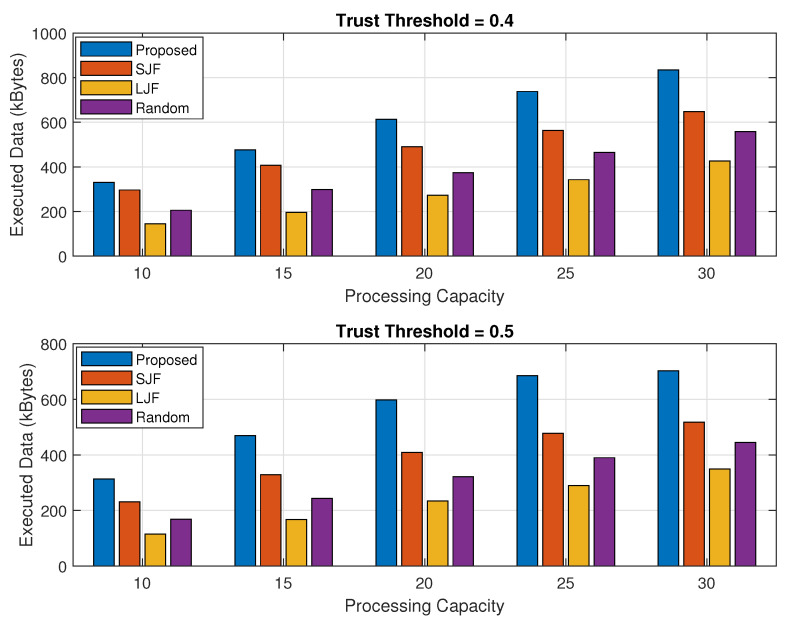
Total executed data against fog processing capacity.

**Figure 10:**
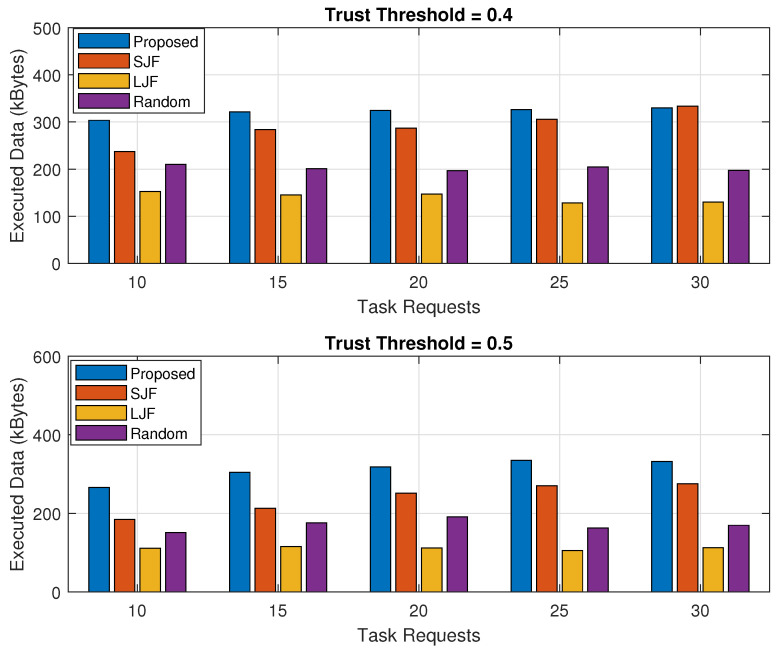
Total executed data against varying number of task requests.

The executed data are calculated by accumulating the amount of data executed by the fog node against all the legitimate task requests initiated by IIoT nodes. The performance of the proposed EEOIT in terms of the executed amount of data for the increasing trend of computing capability of the fog machine and for different amounts of task requests as represented in Figure 9 and Figure 10, respectively. Each of the results is a combination of two sub-results for two threshold values of 0.4 and 0.5. The results of the proposed scheme are compared with other Random, SJF, and LJF.

Results in Figure 9 highlight that the executed data in EEOIT are the highest among all for both the threshold trust values. The results further verify that the amount of data executed by the fog node increases with the increase in its computing capacity. With the trust threshold value of 0.4, when the fog processing capacity is 10, the amount of data executed against legitimate offloaded tasks is 360 kB as compared to 330 kB, 160 kB, and 190 kB of data for SJF, LJF, and Random, respectively. The amount of executed data in the proposed scheme increases to 845 kB as compared to 625 kB, 415 kB, and 585 kB of data in SJF, LJF, and Random, respectively, when the fog computing capacity increases to 30 for the fixed number of offloaded tasks with the threshold value of 0.4. When the threshold value is 0.5, the legitimate task requests decrease and the executed amount of legitimate data by the fog node also decreases. However, executed data in EEOIT are prominently higher than the other competitors. It has been observed from the generated results that the amount of data expected by the proposed scheme is 725 kB as compared to 530 kB, 375 kB, and 430 kB data in SJF, LJF, and Random, respectively,

To validate the performance of our proposed scheme for the executed amount of data, the expected data by the fog machine for increasing the trend of task requests are represented in Figure 10. It is highlighted from the results that threshold trust values of 0.4 and 0.5 are represented in subplots. The results show that for both the threshold trust values of 0.4 and 0.5, when the number of legitimate tasks is more, the amount of data executed by the fog node also increases. When the threshold value is 0.4, the amount of data executed by the fog node in the proposed scheme increases from 300 kB to 330 kB, when task requests rise from 10 to 30 tasks. However, the amount of data executed in SJF, LJF, and Random increased from 250 kB to 310 kB, 160 kB to 130 kB, and 210 kB to 210 kB of executed data, respectively. When the threshold trust value is 0.5, the amount of executed data is reduced as the number of legitimate tasks reduces. The results show that EEOIT due to an efficient TOPSIS-based algorithm executes 350 kB of data as compared to 285 kB in SJF, 110 kB in LJF, and 180 kB in Random algorithms when the number of offloaded tasks is 30 tasks.

### 5.3. Trust Value of Executed Tasks

In this section, the mean trust values (MTVs) of all those tasks that are successfully executed in a specified time are calculated. Suppose *K* task requests are originated by IIoT nodes. If trust value of one of node *i* is calculated as $T_{i}$ then MTV calculated for *k* number offloaded tasks in a sessions as

(10) $$\mathrm{MTV}=\sum_{i=1}^{K}\frac{T_{i}}{K}$$

**Figure 11:**
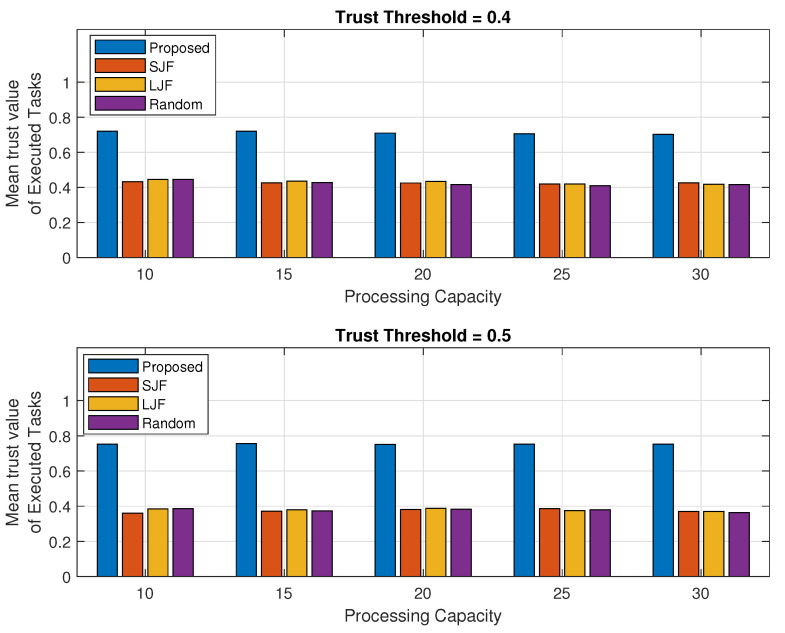
Mean trust value against fog processing capacity.

**Figure 12:**
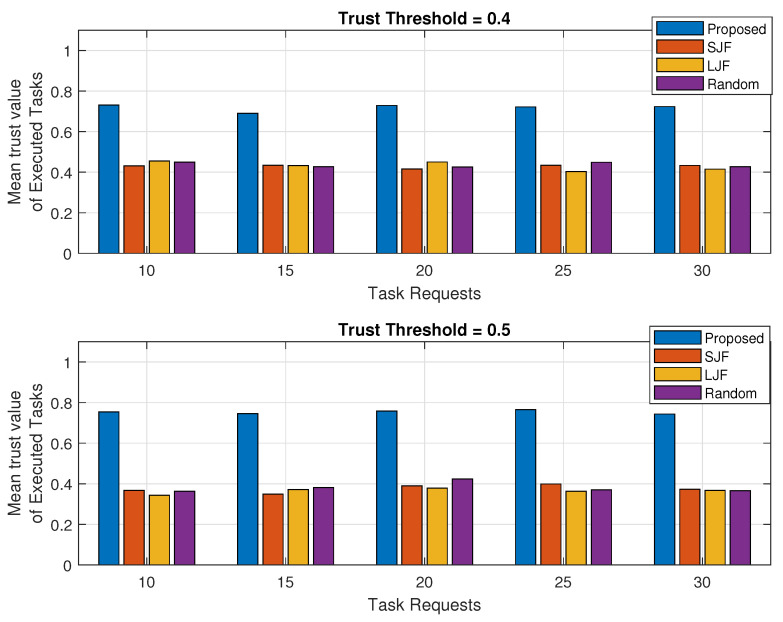
Mean trust value against varying number of task requests.

The MTV is calculated against the executed legitimate tasks for the increasing trend of the tasks processing capability of a fog machine and for different task requests as shown in Figure 11 and Figure 12, respectively. These are also analyzed for two different trust threshold values of 0.4 and 0.5 and represented in each of the sub-plots.

It has been observed that the MTV of the tasks in EEOIT is 0.73 when the trust threshold value is 0.4 and more than 0.78 when the threshold value is 0.5 against all different computing capabilities of fog machines as shown in both sub-figures of the results represented in Figure 11. However, the mean trust value calculated in the other three schemes is about 0.4 for all varying capacities of fog nodes when the threshold value is 0.4 and less than 0.4 when the trust threshold value is 0.5. This huge difference in mean trust value calculations is due to the preference in task selection by applying the TOPSIS algorithm in the proposed scheme. However, SJF, LJF, and Random do not consider the trusted task requests, and some low trust-valued tasks are also executed resulting in reduced mean trust values.

The results in Figure 12 consist of two subplots and represent the MTV of the executed tasks for increasing the trend of requested tasks when the threshold trust value is 0.4 and 0.5. It is evident from the results that the MTV calculated in the proposed scheme is significantly greater than the other three schemes for all different task requests. It has been observed from the results that the MTV calculated in the proposed EEOIT reaches 0.76 when the threshold value is 0.4. However, the MTV calculated in SJF, LJF, and Random is just above 0.4 for all task requests against the same trust threshold value. For the trust threshold value of 0.5, the MTV calculated in the proposed scheme is 0.78 for all originating task requests. However, the MTV calculated in SJF, LJF, and Random is less than 0.4 for all varying amounts of offloaded tasks. This is because, SJF, LJF, and Random execute tasks without considering their trust values. However, in the proposed scheme, preference is given to tasks with higher trust values than others by applying the TOPSIS-based algorithm.

### 5.4. Execution Time

The task execution time is calculated as the accumulated time required to execute all the legitimate tasks. The fog node executes only those offloaded tasks that are within their processing limit. All those legitimate tasks that are not executed by the fog nodes are forwarded to the cloud server with a larger propagation time. Suppose there are a total of legitimate tasks that need to be completed at any given time, which is denoted by $T_{tot}$. Out of these tasks, a certain number of tasks, denoted by *X*, are executed by fog nodes, while the remaining tasks, denoted by *Y*, are executed by cloud servers. If both node *i* with data $D_{i}$ and node *j* with data $D_{j}$ are executed by both fog nodes and cloud servers, then the total time taken to execute all these legitimate tasks can be calculated.

(11) $$T_{tot}=\sum_{i=1}^{X}\frac{D_{i}}{PR_{F}}+\sum_{j=1}^{Y}\frac{D_{j}}{PR_{C}}$$

Here, $PR_{F}$ and $PR_{C}$ represent the data processing rate calculated by the fog node and cloud servers including their propagation delay, respectively.

**Figure 13:**
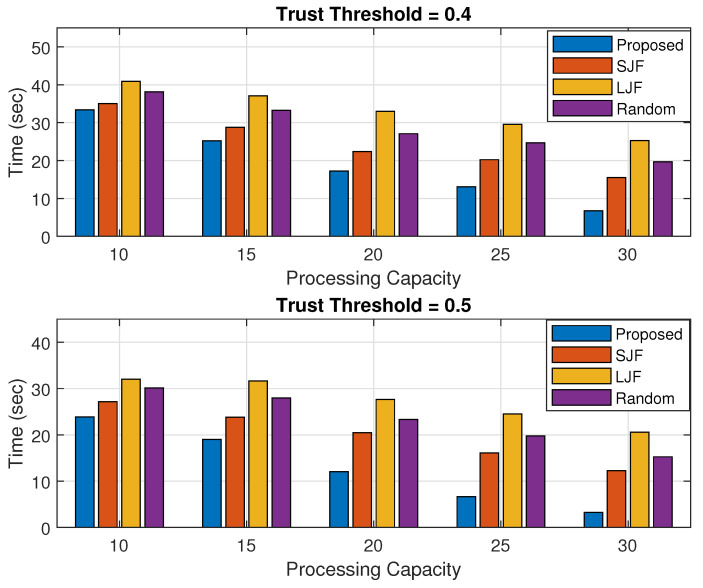
Time against fog processing capacity.

**Figure 14:**
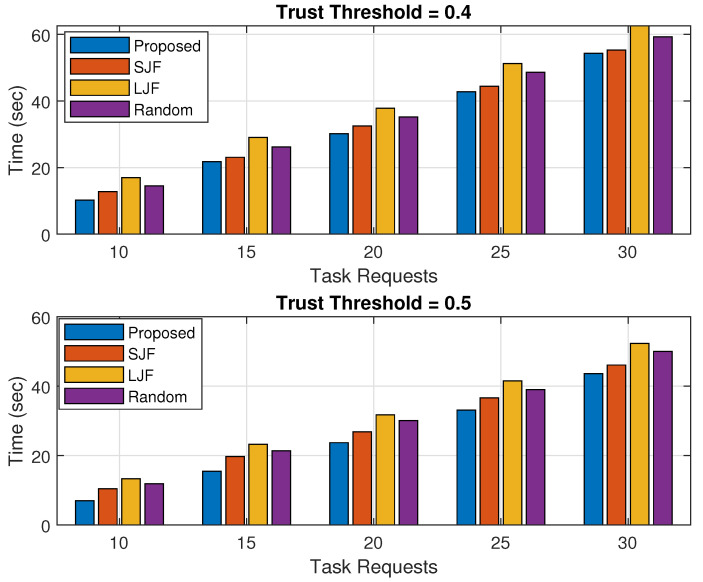
Time against varying number of task requests.

The results shown in Figure 13 and Figure 14 represent the accumulated time calculated in executing all the legitimate tasks offloaded by nodes for varying processing capacity of fog nodes and for varying numbers of offloaded tasks, respectively. The results in each figure are calculated for trust threshold values of 0.4 and 0.5.

The results in Figure 13 show that the accumulated time in task execution in the proposed scheme is less than the other three schemes for both the trust threshold values. This is because the proposed scheme scrutinizes trusted tasks by applying a trust management system. It has also been observed that the task execution time reduces with the increase in processing capacity of the fog node as it can execute more offloaded tasks itself, and lower numbers of tasks are forwarded to the remotely placed cloud server. It has also been observed that for higher trust threshold values, the execution time is reduced as compared to lower threshold values for the same number of offloaded tasks. This is due to the fact that an increased threshold value means there are less legitimate tasks in the total offloaded tasks, and most of these tasks are executed by the fog node with a reduced execution time.

The results shown in Figure 14 verify that the task execution time of the proposed scheme is less than the other three schemes for varying numbers of offloaded task requests. The results show that with the increase in several offloaded tasks with limited fog execution capacity, the task execution time increases for both the trust threshold values. This is due to the reason that only a limited number of offloaded tasks are executed by the fog node and the rest are forwarded to the remotely placed cloud server. The results further show that the task execution time of legitimate tasks is higher for smaller threshold values because in such cases, the number of legitimate tasks increases and the accumulated time in executing these tasks increases.

## 6. Conclusions

The quality of service of the IIoT network is compromised due to malicious node attacks in the network. Malicious nodes create anomalies by compromising the computing capability of the fog machine. In this work, the offloaded tasks are scrutinized by considering their legitimacy. In addition, if the number of offloaded tasks is more than the processing capacity of the fog node, then they are scrutinized by applying the TOPSIS algorithm by considering their trust value, task sizes, and task numbers. The performance of the proposed EEOIT algorithm is compared with Random, SJF, and LJF for different computing capabilities of fog machines and all different numbers of task requests for the trust threshold values of 0.4 and 0.5. It has been observed from the results that EEOIT executes up to 20% more legitimate tasks as compared to SJF, 66% more tasks from Random, and 150% more legitimated tasks as compared to LJF for different computing capabilities of fog machines for both the trust threshold values. The results further show that the EEOIT executes 38%, 65%, and 153% more tasks from SJF, Random, and LJF, respectively, for all varying numbers of task requests. The results clearly show that the executed data of legitimate tasks in EEOIT is up to 32%, 55%, and 164% more than SJF, Random, and LJF, respectively, for both the trust threshold values. It is evident from the results that the execution time of all the offloaded legitimate tasks in the proposed scheme is up to 18% less for varying processing capacity of fog node and 21% less than the other schemes for varying number of offloaded tasks. Similarly, the mean trust value calculated in EEOIT against executed tasks is 68%, 70%, and 73% higher than SJF, Random, and LJF, respectively, when the trust threshold values are 0.3 and 0.5. In future, we will work on improving the trust evaluation metric for different network attacks and efficient load balancing of fog nodes.

## Figures and Tables

**Table 1:** Comparative summary of referenced research.

Ref. No.	Addressed Area	Proposed Scheme	Results
[24]	Trustful communication due to possible malicious issues	Dynamic Trust MATS framework for multiple communication scenarios	Experimental analysis to validate framework
[25]	IoT-based UAV for Intelligent Transportation System	Trust Mechanism by optimizing the trajectory of UAVs to enhance trustable communications	Delay and cost of the system reduced
[26]	Trust challenges of online social networks	Assigned trust value by prioritizing each user	Improved data and trust precision of the network
[27]	Trust management issue in big data	MapReduce-based framework for data processing	Convenience in managing trustable big data
[28]	Online web-based trust challenges in virtual network during its reboot	Prototype based on open-source MANO platform	Performance improvement in dynamic environment
[29]	Explored an IoT-based healthcare system with decentralized trust management system	Evidence–theory reliant solution with reward and punishment system	Robustness and security enhancement
[30]	Easy and confidential intervene of technicians in industry	Software architecture to simplify the management, configuration and assessment	Experimented in railway use case
[31]	Management issues by data owners authority due to third party	Blockchain-based data sharing system by applying BAN logic	Guaranteed secure mutual authentication
[32]	Real-time security issues in IoT network	Layered architecture in fog computing paradigm	Real-time security issues are resolved

**Table 2:** Simulation environmental parameters.

Parameter	Value Range
Coverage Area	10 × 15 = 150 m${\;\;}^{2}$
Offloaded Tasks in each Time Interval	10:2:20
Size Range of Offloaded tasks (kBytes)	60 to 120
Computing Capability of Fog machine (kB)	300
Computing Cycle of Fog Node in each time interval	10
Deadline of Requested Tasks (Processing Cycles)	10 to 20
Malicious nodes	1 to 2
Legal nodes	9 to 18
Trust Threshold	0.4, 0.5

## Data Availability

Data are contained within the article.
